# In Situ Micro-Observation of Surface Roughness and Fracture Mechanism in Metal Microforming of Thin Copper Sheets with Newly Developed Compact Testing Apparatus

**DOI:** 10.3390/ma15041368

**Published:** 2022-02-12

**Authors:** Mandeep Singh, Shubham Sharma, Appusamy Muniappan, Danil Yurievich Pimenov, Szymon Wojciechowski, Kanishka Jha, Shashi Prakash Dwivedi, Changhe Li, Jolanta B. Królczyk, Dominik Walczak, Tien V. T. Nguyen

**Affiliations:** 1 School of Mechanical and Mechatronic Engineering, University of Technology Sydney, Ultimo, NSW 2007, Australia; 2 Department of Mechanical Engineering, Main Campus-Kapurthala, IK Gujral Punjab Technical University, Jalandhar 144603, Punjab, India; 3 Department of Mechanical Engineering, University Centre for Research and Development, Chandigarh University, Kolkata 140413, Punjab, India; 4 Department of Automobile Engineering, Saveetha School of Engineering, Saveetha Institute of Medical and Technical Sciences, Chennai 600124, Tamil Nadu, India; inspire.munish@gmail.com; 5 Department of Automated Mechanical Engineering, South Ural State University, Lenin Prosp. 76, 454080 Chelyabinsk, Russia; danil_u@rambler.ru; 6 Faculty of Mechanical Engineering and Management, Poznan University of Technology, 60-965 Poznan, Poland; sjwojciechowski@o2.pl; 7 Department of Mechanical Engineering, Lovely Professional University, Delhi 144001, Punjab, India; kanishkaero@yahoo.com; 8 G.L. Bajaj Institute of Technology and Management, Greater Noida, Gautam Buddha Nagar, Greater Noida 201310, Uttar Pradesh, India; spdglb@gmail.com; 9 School of Mechanical and Automotive Engineering, Qingdao University of Technology, Qingdao 266520, China; sy_lichanghe@163.com; 10 Department of Manufacturing and Materials Engineering, Faculty of Mechanical Engineering, Opole University of Technology, Mikolajczyka 5, 45-271 Opole, Poland; d.walczak@po.edu.pl; 11 Department of Industrial Engineering, Industrial University of Ho Chi Minh City, Ho Chi Minh City 70000, Vietnam; thanhtienck@naver.com

**Keywords:** universal testing machine (UTM), metal microforming, copper samples, tensile test, 3D laser-confocal microscope

## Abstract

A better understanding of material deformation behaviours with changes in size is crucial to the design and operation of metal microforming processes. In order to facilitate the investigation of size effects, material deformation behaviours needed to be determined directly from material characterizations. This study was aimed at the design and manufacture of a compact universal testing machine (UTM) compatible with a 3D laser-confocal microscope to observe the deformation behaviour of materials in real-time. In this study, uniaxial micro tensile testing was conducted on three different thin (0.05 mm, 0.1 mm, and 0.3 mm) copper specimens with characteristic dimensions at micro scales. Micro tensile experimental runs were carried out on copper specimens with varying grain sizes on the newly developed apparatus under a 3D laser-confocal microscope. Microscale experiments under 3D laser-confocal microscope provided not only a method to observe the microstructure of materials, but also a novel way to observe the early stages of fracture mechanisms. From real-time examination using the newly developed compact testing apparatus, we discovered that fracture behaviour was mostly brought about by the concave surface formed by free surface roughening. Findings with high stability were discovered while moving with the sample grasped along the drive screw in the graphical plot of a crosshead’s displacement against time. Our results also showed very low mechanical noise (detected during the displacement of the crosshead), which indicated that there were no additional effects on the machine, such as vibrations or shifts in speed that could influence performance. The engineering stress-strain plots of the pure copper-tests with various thicknesses or samples depicted a level of stress necessary to initiate plastic flowing inside the material. From these results, we observed that strength and ductility declined with decreasing thickness. The influence of thickness on fracture-strain, observed during tensile testing, made it clear that the elongation-at-break of the pure-copper foils intensely decreased with decreases in thickness. The relative average surface-roughness R_a_ was evaluated, which showed us that the surface-roughness escalated with the increasing trend of plasticity deformation (plastic strain) ε. For better understanding of the effects of plastic strain on surface roughness prior to material fractures, micro tensile tests were performed on the newly developed machine under a 3D laser-confocal-microscope. We observed that homogeneous surface roughness was caused by plastic strain, which further formed the concave surface that led to the fracture points. Finally, we concluded that surface roughness was one of the crucial factors influencing the fracture behaviour of metallic sheet-strips in metal microforming. We found that this type of testing apparatus could be designed and manufactured within a manageable budget.

## 1. Introduction

In this modern world of miniaturization, there is an enormous need for microscale metallic components for industrial applications [1]. The directional/geometrical accuracy and reliability of such microscale metallic parts has been estimated to be in the range of 0.1 µm to 10 µm [1,2]. In recent years, various advanced micromanufacturing processes have been invented, and they have been continuously used to manufacture microscale metallic parts such as connectors pins, micro screw and springs [3,4]. The standard micromanufacturing processes are shown in Table 1. Metal microforming is an effective process for large-scale manufacturing of intricate and high-performance microscale metallic segments [5]. Metal microforming is an excellent process because of its notable benefits (for example: high creation rates, close net shapes, limited material waste, and close tolerance) [5].

In a renowned study [7], metal microforming was described as a standard microscale-deformation procedure used to create sub-millimetre extended metallic parts with multi-assistance. In another work [8], all other micromanufacturing methods, as options in microscale metal forming, were briefly studied; however, it was concluded that the metal microforming process was a promising approach as it had the ability to create an enormous volume of microscale metallic parts cost-effectively. These highlights make metal microforming an appropriate process for the high-volume creation of metallic parts with desirable toughness and quality with minimal effort [9]. Metal microforming is a prominent area of research in which size effects can play a leading role. In microscale metal forming, all procedural issues and material conduct issues that grow more complicated as they are scaled down are major sources of concern with respect to the impact of size [10]. In other words, size effects arise when the proportional extents of a specimen are changed, and the ratio among certain characteristics cannot be reserved constantly [11]. In all microforming processes, the material deformation and surface roughness issues are directly affected by size [12]. Therefore, all of these issues need to be concerned continuously with metal microforming operations in order to reach the required dimensions in final products.

Surface roughness is one of the key problems in metal microforming [13]. In metal microforming, the surface roughness does not decrease with the decline in foil thickness [13]. In a recent study [14], researchers concluded that the expansion in surface roughness of the metal foils happened during plastic deformation, which led to further fracture behaviour. In metal microforming, surface roughening occurs easily on the free surface of the specimen, since the distortion testing comprises a multiple-granule particulate [15]. The surface roughness occurs mainly due to crystal orientation among the grains during the metal microforming processes. Differences in thickness and surface abnormalities can be attributed to the differentiation of the individual grains’ Schmid factor, which in turn determines the slip mechanism for the occurrence of surface roughness [16]. In most previous studies [15,16,17], surface roughness and fracture behaviour were inspected in the context of the cumulative impact of work-piece geometry and granularity particle size, which demonstrated that the surface harshness of the twisted sample increased with the diminishing of t/d. This can be attributed to the fact that surface granules are less obliged and more easily misshapen on free surfaces with lowT/D. However, in a few studies, the effects of foil thickness on surface roughness were examined. Notably, in previous studies [18], the effects of plastic strain on surface roughness and fracture behaviour were not investigated in situ. Moreover, prior investigations have been constrained uniquely to sheets with a thickness of more than 0.5 mm.

Therefore, the target of this work was to assess the free surface roughening in metal foils with different thicknesses and to further explore the impacts of surface harshness on fracture behaviour in real-time. To facilitate this, the testing system should have the ability to perform mechanical tests on miniature (i.e., extremely small and thin) specimens. To achieve this objective, a new compact testing instrument was developed in this study. Over the past few years, the development of miniature testing apparatuses has attracted the interest of researchers. However, established systems [19] were deemed overly complicated and expensive. To understand and optimize the development of a miniature testing apparatus, it was necessary to review some basic scientific facts first. Therefore, detailed analyses of selected aspects of intensive literature concerned with miniature testing apparatuses were briefly discussed and studied to evaluate their possible mechanisms. After briefly reviewing previous studies [20,21], we determined that it was essential to develop a new, highly precise compact testing apparatus compatible with a 3D laser-confocal microscope. The main reason behind this was the need for in situ observations to aid in checking and understanding all functions acting within the system and effect required changes. There were some compact size testing machines available in the current market, but our needs still called for enhancing enhance the efficiency of the testing system, for instance by reducing its overall weight. A high degree of reliability is an essential characteristic for any engineering systems, and these new types of compact testing instruments are no exception [22]. Currently, all compact-sized instruments require a substantial number of microscale components such as miniature screws. These micro-level connecting parts can be created using metal microforming methods on a thin (5 m–100 m) sheet; however, traditional microforming processes do not work in this thickness range. Because the mechanical properties of a sample are altered during microforming (due to the reduction in sample dimensions), so-called size effects occur.

Following a study of prior investigations, it was determined that the material behaviour in microforming is impacted, not only by overall dimension, but also by microstructural topographies, most notably grain size. As a result, to permit a thorough understanding, both aspects (workpiece thickness and grain size) were included in this work. Furthermore, a ratio of sheet thickness to average grain-size ((T/D) > 1) was used in this study for flow stress analysis. To examine the significance of T/D on flow stress, the selected T/D range was less than 3. With these models, researchers described the implications of size on distinct material deformation behaviours in microforming. To fit the experimental data, all of the models directly incorporated dimension components, such as T and D, into the standard constitutive model. In this novel work, some modifications were proposed in a material model in order to predict the correct trend of flow stress.

In this article, a special phenomenon in metal microforming processing called size effects was studied. Furthermore, experimental investigations and numerical studies on material deformation behaviours were discussed in detail. All of the researchers conducted parametric research and concluded that the advancement of metal microforming processes could be characterized accurately by the measurement of their properties. However, the investigators also identified critical issues like size-effects in the metal microforming system.

Following studies from past research, it was discovered that, in metal microforming processes, the grain size size effect plays a more significant role than other factors, and that the function of T/D is a critical condition to distinguish microscale from macroscale. Due to the important role of grains in microscale processes, the strength of the material can be determined by measuring changes in average grain size. This assertion was based on the fact that granular boundaries hinder dislocation mobility; thus, the number of deformations inside a grain tends to increase. As a result, altering the grain size can affect movement of dislocations and enhance the materials’ characteristics. Heat treatment after plastic deformation and altering the solidification rate are two of the alternative approaches to changing particle size.

There are some gaps in the research of metal microforming; for instance, the relationship between grain size effect ratio and deformation behaviour, especially when the ratio is limited in a certain range. A more accurate modelling technique for FE simulation is required. No significant research has been done on the plasticity model of microbending, with characteristics’ length to capture size effects. In situ observation of surface roughening during plastic deformation is required for a better understanding of the influences of size effects.

Base on the above summary and analyses, in this study, the following objectives were set, with the aim of obtaining a clear and unambiguous understanding of size effects in metal microforming: (1) to determine how size effects impact flow stress in terms of a given specimen’s thickness/average grain size (T/D) in polycrystalline structures; (2) to propose modifications to material models in order to describe size effects in polycrystalline structure and deliver reliable calculations; (3) to determine how T/D ratios affect the evolution of surface roughness; (4) to develop a new micro-testing machine (UTM) compatible with 3D laser-confocal microscopes for in situ micro-observations.

A compact UTM, compatible with a 3D laser-confocal microscope, was successfully developed and applied to the task of observing the deformation behaviour of materials in real-time. However, there were some drawbacks to the newly invented equipment—i.e., grain orientation has an obvious effect on elastic and plastic deformation behaviours of the metal. Its effects on springback can be studied in the future. Grain orientation information in three different T/D materials could be obtained by electron backscatter diffraction (EBSD) technology, and the crystal plasticity finite element method (CPFEM) could be adapted to explore the effects of size on springback. To understand the relationship between surface roughness and technical parameters such as grain size, a constitutive model should be generated to simulate the surface evolution. New testing apparatuses could be used with a 3D laser-scanning microscope to evaluate the deformation behaviours of other materials and miniaturised components. The developed apparatus is not only suitable for metals; it could also be used to test ceramics, composites, and polymers. In biomedical engineering, newly designed and developed bone plate fractures could also be examined in detail with the help of this apparatus. Moreover, due to the novelty of this new compact testing apparatus, there is a potential for patent submission and commercialization. 

To represent the hardening behavioural patterns of granularity in polycrystalline material, a far more effective material model was constructed, such that the model could only examine the deformation behaviour up to a few points before necking. The strain-gradient must always be computed/evaluated by strain-gradient tensor operations during the evaluation of the microbending operation of metallic foils. The constructed instrument could also be utilised for compressive and bend measurements with required fasteners/attachments and specimens. However, these challenges were not addressed in this work in order to confirm the validity of the developed apparatus.

This study focused on the micro tensile test because it is a significant microforming process, and ideal for the testing and manufacture of microscale metallic parts. In addition, to verify the performance of the developed machine, the reported results of thin copper samples were compared with the outcomes of comparable samples performed in a commercially available UTM, METEX—1 kN, using similar crosshead speeds.

## 2. Development of New Compact Testing Apparatus

Metal microforming is a suitable and relevant method for the manufacture of microscale metallic parts. With the fabrication of microscale parts, it is essential to analyse the engineering design, analysis for which the stress-strain relationship is crucial. Consideration of the numerous mechanical characteristics of both of the specific materials (including ultimate and yielding strengths, elastic modulus, and the Poisson’s ratios) encompasses the study of the stress-strain relationship for small-scale samples. The tensile test is the most significant method used for determining a material’s stress-strain relationship. It has been broadly used due to its high degree of flexibility and its economic advantages. The universal testing machine (UTM) is one of the most common pieces of equipment used for tensile testing. This type of traditional testing machine is relatively heavy and is typically installed in a laboratory. In order to evaluate mechanical properties, these machines require relatively large material samples. In traditional testing machines, two crossheads are used; one to adjust the length of the sample, and the other to apply force to the test sample. Traditional testing machines are inefficient at applying moderate force/pressure on smaller tested specimens in order to adequately reproduce actual-force applications. It is difficult to assess the strengths of small (microscale) samples on a traditional UTM. The relationships between dimensions and surface geometry in treated workpieces, as well as in tools, are different in macro- and microscale, which can directly affect the stress-strain relationship. Furthermore, surface roughness is one of the core problems in metal microforming, mainly caused by non-uniform deformation of metal foils. Increased surface roughness of metal foils occurs during plastic deformation—which was investigated in this work. Thus, it was deemed important to investigate the mechanisms of surface roughness in metal microforming process in real-time to enable better analysis. Finally, it was concluded that in situ observations of the deformation behaviour and surface roughness phenomena of different materials foils would be a great help in efforts to understand the influence of size effects in metal microforming. To permit this, the testing system needed to have the ability to perform mechanical testing of miniature (i.e., extremely small and thin) specimens. Over the past few years, the development of miniature testing apparatuses has attracted the interest of researchers. Currently established systems are very complicated, and only a few innovative small test machines are available in the market. However, even regarding available machines, budgetary limitations are another common barrier to acquiring such apparatuses.

As a result, a novel, highly precise, efficient compact testing apparatus was required. In this study, a novel, compact, portable UTM with highly precise driving systems and higher accuracy loading and displacement measurements was designed. It was intended for numerous mechanical testing experiments with optimum data acquisition, control capabilities, and effectiveness. The primary intention was to establish an innovative, revolutionary compact portable miniature UTM that could be coupled with a 3d laser confocal-scanning microscope for in situ micro-observations of size effects. With the appropriate fastener attachments and specimens, the designed apparatus could also be employed in compressive as well as bend testing measurements; however, these challenges were not addressed in this study.

Once the machine was developed, metallic test samples made of thin copper sheets were used for tensile testing. To verify the performance of the developed machine, the reported results were compared to the outcomes of comparable test specimens in a commercialized UTM (METEX—1000 N) at similar crosshead speeds.

**Figure 1:**
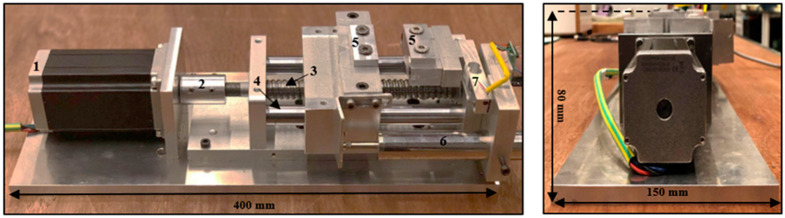
Overall structure of the testing machine: (1) stepper motor, (2) coupling, (3) lead screw, (4) supported columns (5) tensile test fixtures, (6) LVDT, (7) load cell.

**Figure 2:**
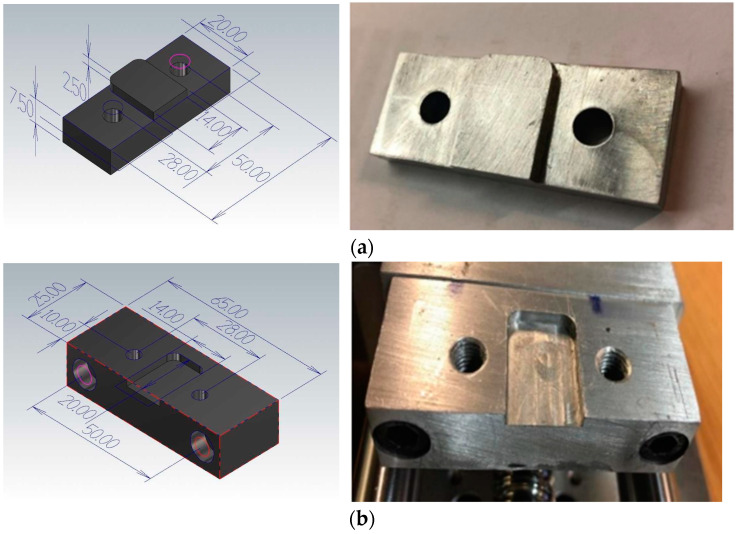
(**a**) Male part of tensile fixture, (**b**) Female part of tensile fixture (units: mm).

In recent studies [23,24], the action of surface roughening before fracture was not easily observed, since fracture happens quickly. In this paper, a newly-made compact testing apparatus for in situ micro-observation was used. Figure 1 shows the overall structure of the established testing machine, with a total length of 400 mm, width of 150 mm and height of 80 mm. The developed instrument was designed to test small and thin specimens, including metals, polymers and metallic alloys, in the selected load range. The device is proficient at analysing tensile samples up to 8 cm of length. Table 2 shows all the specifications of the developed apparatus. This instrument was mechanically designed to minimize the influences of loading incorporation in the mainframe and relative motion among the portable crossheads. The measuring system was primarily composed of aluminium alloy, excluding certain frictional components, such as the bearing and ball screw, which are made of steel. Upon reviewing past research [23,24] and common standardized test methods [25], we discovered that screw-driven machinery has been prominently used in construction of ideally compact UTMs. Therefore, a small CNC linear table, driven by a ball-screw, was selected. The specimen holder consisted of two fixtures made of aluminium, as shown in Figure 2. The tensile test fixtures were provided in the form of male and female parts to uniformly press the samples in order to carry out the actual testing without slippage. This portable UTM consisted of a stepper motor, a load cell, an LVDT, a load cell amplifier, and a data acquisition system. For quantifying loads and percent elongation at the same time, a novel Arduino code program was developed and transferred to a data acquisition device (Arduino). The Tera-Term program was employed to send the Arduino sensor information to an Excel spreadsheet. With this process, all the parameters (applied force, displacement, and time) were captured and saved in real-time for subsequent examination. The general features of the developed machine are described herein.

### Features

- Oil-free device for clean conditions.
- Easily-controlled system (e.g., emergency stop).
- Ability to test miniaturized components (e.g., strain gauges).
- No hydraulic or pneumatic air supplies.
- Compatible with 3D laser-confocal microscope.

**Figure 3:**
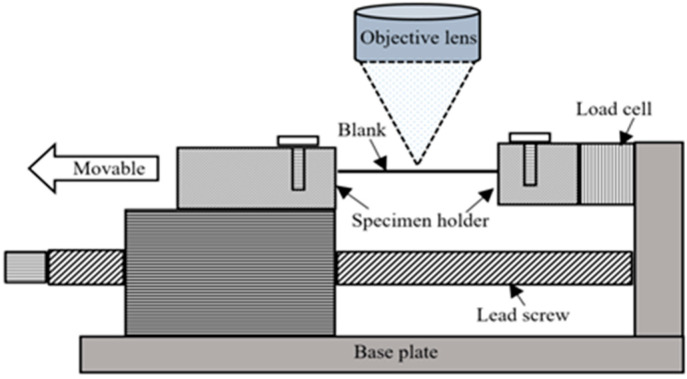
Schematic of the experimental test assemblage.

**Figure 4:**
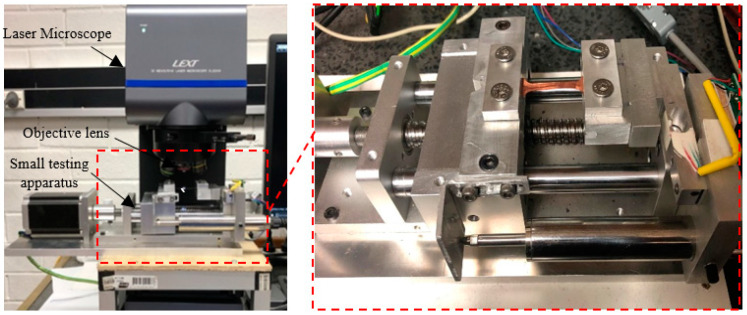
The testing apparatus under laser microscope.

In this small UTM, a stepper motor (NEMA 23 Stepper Motor 3 Nm) was used to drive the linear table with ball-screw guideway. It was crucial to monitor both the speed and position of the retractable clamping fasteners for various testing activities; hence, a stepper motor was used. The die and blank holder were moved by one revolution of the handle at 5 mm displacement. Therefore, to measure the material strain more precisely, the motion of the crosshead displacement of the device was measured with a precision digital displacement gauge (LVDT). Additionally, the crosshead displacement was assumed to be equal to the change in gauge length. The apparatus was very compact in size, enabling its placement under the 3D laser-confocal microscope, as shown in Figure 3. During in situ observation, a huge region of the extended blank surface was observed by 3D laser-scanning microscope with a high magnifying lens (5×: 0.45 μm). In addition, even with other high magnification lenses, it was possible to detect a wide area of the stretched blank surface. A constant distance was maintained between the extended surface and the reference targeted lens throughout analyses. The compact, portable testing apparatus was fabricated with an extremely precise drive system and high accuracy load and displacement measurement functions in order to carry out tensile testing with the utmost control and data collection performance. Figure 4 shows the schematic of the experimental test assemblage structure.

## 3. Experimentation

**Figure 5:**
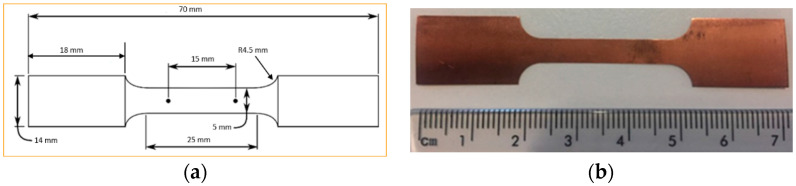
(**a**) Schematic of micro tensile samples. (**b**) Schematic of actual copper specimen.

Pure copper (99.9%) flakes with thicknesses, t, of 0.05, 0.1 and 0.3 mm, respectively, were utilized in this examination. The chemical content of the pure copper is displayed in Table 3. Figure 5 shows the dimensions of tensile samples. The dimensions of the tensile sample were designed according to ASTM E8/E8M. After cutting all the samples into a bone shape, they were annealed for 10 min at 700 °C in order to achieve an effective blend of physicomechanical characteristics. All the micro tensile samples were annealed in an NBD-O1200 vacuum tube annealing furnace. Since the specimens were so compact, Argon air shielding was used in addition to a vacuum environment throughout thermal treatment to prevent oxidation.

The challenging part of this work, after annealing, was achieving a high-diamond finish on copper specimens in order to facilitate measuring the average grain size. The copper specimens were polished with fine Alpha aluminium powder (0.3 and 0.5 μ). The Alpha aluminium powder was continually pressed upon the specimens throughout polishing to produce an effective shine on the softer copper materials. To ensure a fine level of flatness during polishing, all the copper samples were mounted on smooth, round pieces of hard plastic (Polyfast powder). Both grinding and polishing were performed on the Struers automatic Tegrapol 21 machine. The grinding and polishing trials for metallography are exhibited in Table 4. Afterward, for microstructure analysis, the samples were treated further. The line intercept method was used to measure the average grain size. A micrograph (microscopic photograph) of one copper sample was used to observe the average grain size.

**Figure 6:**
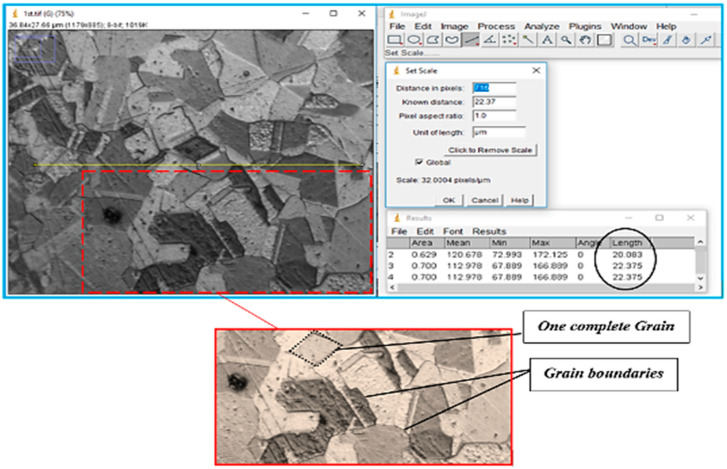
Optimization of average grain size in ImageJ software.

The microstructural image of the copper sample was moved to ImageJ software to measure the actual grain size. In Figure 6, the line segment (yellow line) that is randomly placed over on micrograph shows the first step in determining average grain size. In ImageJ, the microstructure picture was changed to a grayscale image, as shown in Figure 6, because the grain boundaries were darker than the grains themselves, and grayscale made it easy to count the grain boundaries intersected by the line segment. Table 5 shows the calculated number of grains, nG, in the direction of thickness and the average grain size, d.

**Figure 7:**
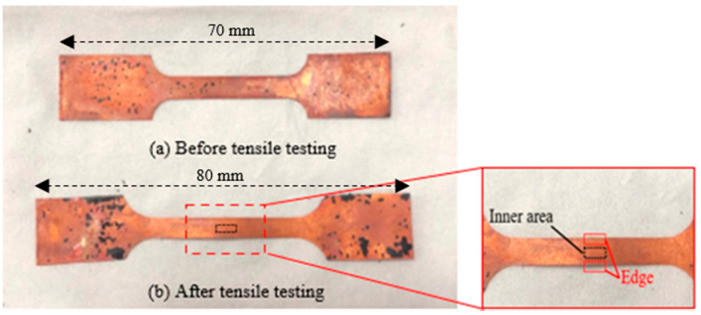
Real copper samples.

**Figure 8:**
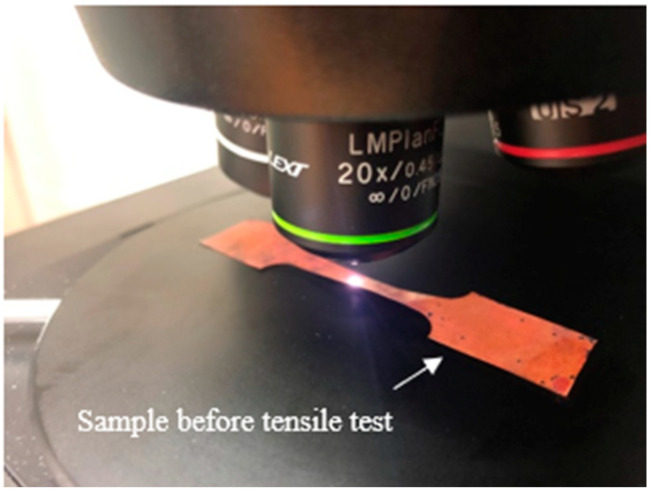
Surface roughness testing.

The bone-shaped copper specimens, before and after micro tensile testing, can be seen in Figure 7. The edge thicknesses of both tested and untested samples were lower than the thicknesses of the interior portions. Therefore, observation of the surface roughness was obtained from the inner areas of the tested samples. The surface roughness was evaluated in the selected region (black area) using a 3D laser-confocal microscope, as shown in Figure 8. The roughness profiles, waviness profiles and 3D surface textures of both tested and untested samples were used to check the surface roughness. All the tensile tests were performed on UTM with the maximum capacity of 1 kN and crosshead velocity of 0.09 mm/s. In the developed apparatus, the specimen holders, or grippers, were designed to hold the bone-shaped test samples without slippage in order to carry out the actual tensile testing. Each fixture consisted of a fixed part and a movable plate joined with two screws to press the samples uniformly. One specimen holder was attached to the load cell for monitor loading durations, while the second clamp was rotated to the front and back by a lead-screw-nut arrangement to generate significant pressure. The material strain was measured by movement of the machine’s crosshead displacement using an effective digital displacement gauge (LVDT) with a resolution of 0.01 mm and a maximum displacement of 20 mm. For accuracy, all the experiments were conducted 4 times.

## 4. Results and Discussions

**Figure 9:**
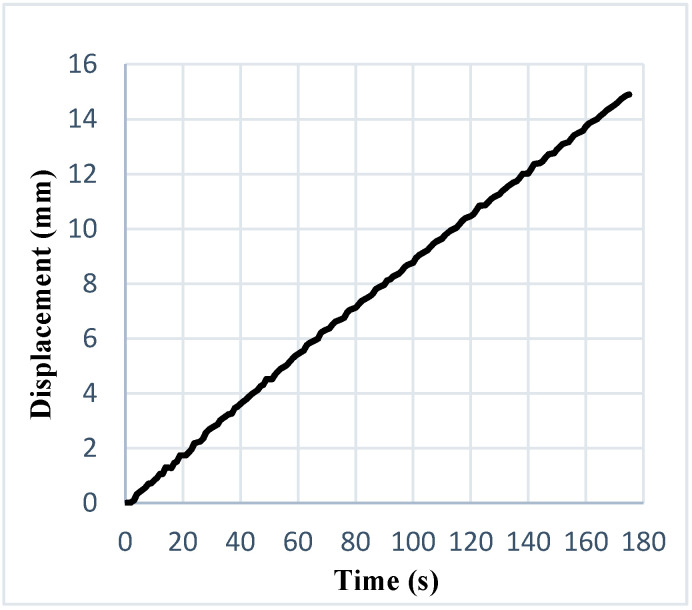
Displacement vs time graph.

To validate the developed testing machine, a pure copper foil with a thickness of 50 μm was selected for the actual tensile tests. All the tensile tests on the new UTM were performed with the same crosshead velocity, so that the results and performance of the newly-developed machine could be compared with reported test results derived from a standard testing machine. Figure 9 displays a plot of the moving crosshead’s displacement against time, wherein high stability could be found while moving with the sample grasped along the drive screw. Figure 9 also illustrates the very low mechanical noise observed during the displacement of the crosshead, which means that there were no additional effects on the machine, such as vibrations or shifts in speed, that could influence performance. The equipment offered ultra-precise displacement control.

**Figure 10:**
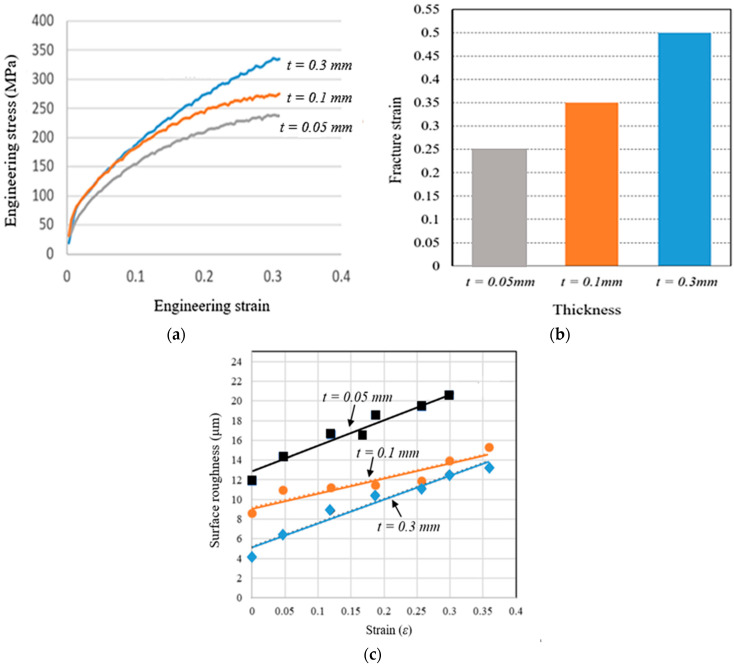
(**a**) Influence of thickness on stress-strain, (**b**) influence of thickness on fracture strain, and (**c**) influence of strain on surface roughness.

This instrument provided very accurate stress-strain measurements; it is highly suitable for investigations of microscale-level deformation behaviour. The impact of the sample thickness was very significant, specifically when the specimen was thin. The engineering stress-strain plots of the pure copper tests with various thicknesses or samples are shown in Figure 10a–c. The stress-strain diagrams depict a level of stress necessary to initiate plastic flowing inside the material. From these results, it can be observed that strength and ductility declined with decreasing thickness. To calculate the relative surface roughness behaviour of pure copper foil, micro tensile testing was performed on the newly-developed machine under a 3D laser-confocal microscope. The arithmetical mean of the roughness profile, R_a_, was evaluated. All the tests were repeated four times.

To examine the impact of flow stress and material qualities on size, Suzuki et al. [26] revised the tensile and compression tests. Flow stress and flow curves were used in metal forming to characterize the material behaviour. Measurements of flow stress in various tensile test settings of different materials were carried out to examine the specimens’ sizes and their effect (to) on the material behaviour. Based on these findings, it can be inferred that, when the length scale changes from macro to micro levels, grain size has a more significant impact on flow stress and material behaviour, rather than specimen size [27]. According to most studies, when specimen size decreases, the flow stress decreases as well, which is caused by an increase in the inclination share of the surface grains. Even though T/D condensed to range 24, Anand et al. [27] showed an increase in flow stress in various material tests. For instance, a decrease in the T/D value from 3.9 to 3.2 was detected in tensile tests of 99.999 percent Al and bulging tests of CuZn36 as an increase in flow stress. When the T/D ratio (single crystal deformation) for CuZn15 was lowered to 1, an upward trend in flow stress was also observed [28].

Sutou et al. [29] used tensile testing on different sized Cu sheets in order to find out how the materials’ behaviour changed with sheet size. These changes were a result of the diminishing grain boundary strengthening effect. Wang et al. [30] conducted experiments and discovered that uneven material flow first appeared near the materials’ extremities rather than their centres. Coarse-grained samples showed inhomogeneous and anisotropic material behaviour, and the ratio of sample size to grain diameter grew increasingly accurate as sample size increased. Because of the variations in grain size, shape, and orientation caused by inhomogeneous billet deformation, deformed samples had non-uniform forms and deformation processes.

The influence of thickness on fracture-strain acquired during tensile-testing is shown in Figure 10b. From this figure, it is clearly evident that the elongation-at-break of the pure copper foils intensely decreased along with decreasing thickness. Figure 10c shows the relative surface roughness behaviour of pure copper flakes with various thicknesses obtained from micro tensile tests.

**Figure 11:**
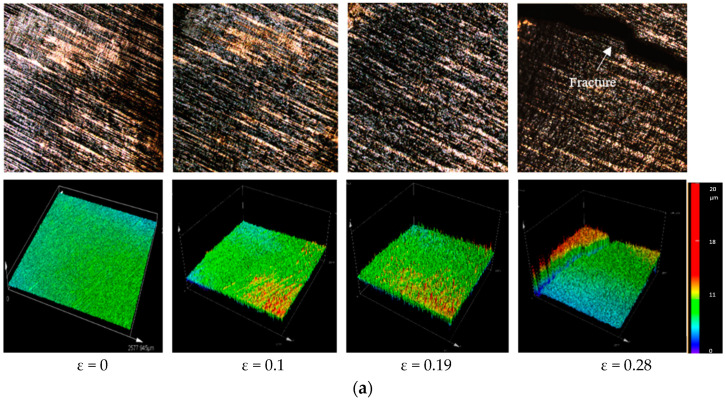

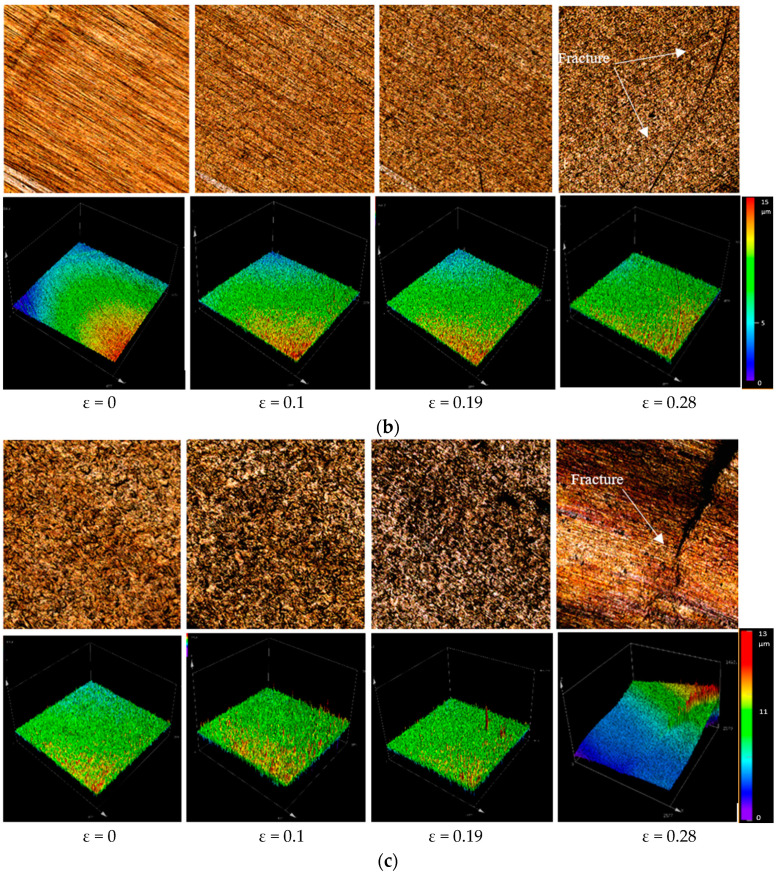
(**a**) 3D surface profile of copper foil (t = 0.05 mm) obtained from laser microscope. (**b**) 3D surface profile of copper foil (t = 0.1 mm) obtained from laser microscope. (**c**) 3D surface profile of copper foil (t = 0.3 mm) obtained from laser microscope.

In Figure 11a–c, the relative average surface-roughness (R_a_) is evaluated. The surface-roughness escalated with the increasing trend of plasticity deformation (plastic-strain) (ε). The slopes of surface roughness were different for all thicknesses and a significant change was observed in surface roughness slope when the thickness changed from 0.05 to 0.3 mm. To allow better understanding of the effects of plastic strain on surface roughness prior to material fracture, micro tensile tests were performed on the newly-developed machine under a 3D laser-confocal microscope. The surface roughness was estimated at the same area in four stages using a high magnifying lens. The evaluated three-dimensional surface profiles of tested samples are shown in Figure 11a–c. From these three-dimensional surface profiles, it was observed that homogeneous surface roughness was caused by plastic strain, which further formed the concave surface that led to fracture points. Finally, we also concluded that surface roughness a crucial factor influencing the fracture behaviour of metallic sheet strips in metal microforming.

The Schmid law states that shear stress is greatest when it occurs in the slip plane and direction [31]. The primary cause of grain distortion is crystal slide or slipping. Because of this mechanism of deformation, a single grain has anisotropic characteristics. Due to the large number of grains in macroscale polycrystalline materials, the grains might be dispersed uniformly or randomly and have diverse properties. In contrast, with fine grain sizes, samples are made up of many small particles and no longer have a distinct distribution of grains. Anisotropy should alter flow behaviour and lead to inhomogeneous deformation if every grain has it. There is no denying that fracture behaviour differs greatly between micro and macro formations [32]. To better understand how metal microforming fracture behaviour changes with increasing size, researches have performed a variety of experiments and simulations. For a given grain size, Furushima et al. [33] looked at the fracture behaviour of brass in a compression test and found that formability increased as specimen sizes were reduced. It is also known that greater strain or deformation is required to create a fracture in micrometals.

Fracture strain reduces in proportion to specimen size, just like flow stress [34]. This is the case in metal compression, where greater strain is required to reach critical damage energy. There have also been investigations into the relationship between fracture strain and changes in T/D in tensile tests. In one study, fractography was investigated on a tensile-tested sample with two distinct separation thicknesses (600 and 100 m) and varying T/D values. It is worth noting that different fractography images have varied thicknesses and average grain size ratios. As T/D decreases, so do fracture strains and micro-voids on the fracture surface [33]. Metal microforming processes exhibit fracture behaviour that is mostly caused by the small grain size of thin metal sheets. In metal microforming, due to the reduction in grain boundary shares when a sample is only a few grains thick, voids can occur [33].

As T/D decreased, so did the number of micro-dimples and micro-voids. When the thickness was only one grain, there were no micro-voids (T/D = 1.2). Using fractographs, we observed that the material could exhibit a mixture of ductile and brittle fracture modes, reflecting unique properties in the three different T/D values. In contrast, when T/D = 1, only a few micro-voids could be found, leading to a brittle fracture nature, and only a few micro-dimples were observed concentrated in a small area. Materials tend to fracture in a ductile mode when T/D = 1 because micro-voids are evenly distributed along the fracture direction. To perform DCE, workpieces with scaled-down diameters between 4.8 and 5.0 mm can be used [35]. If the height of the two cups in a double extrusion process are the same, the friction will be minimal. Another interesting fact about the double cup extrusion process is that it begins with maximum friction and then lowers slightly before increasing again at the conclusion. Micro-sheet formation was found to be more friction-sensitive by Eckstein et al. [35] than micro-bulk formation. In terms of determining friction coefficients, the strip drawing approach is the quickest and easiest option. For microforming, the friction factor m is higher than for macroforming, as reported by Vollertsen et al. [36]. They developed a new friction test model and found that this was the case (μ = *F*2*N*, where F and N are the friction force and normal force, respectively). It is apparent from most experimental research [37,38] that the friction coefficient increases (with lubrication) with a smaller specimen size. An alternative method [39], instead of the deep drawing process, was utilised to identify tribological size effects, taking into account the tangential pressures that exist when a blank is drawn into a die. Additional modification is necessary to create numerous nanoscale crystals on the sample surface, which then trap the lubricating oil and reduce friction [40]. In metal microforming, the consequences of the die-to-workpiece interface are crucial. According to Geiger et al. [37], friction can contribute to incorrect metal microformation outcomes. Because their deformation sample contained multiple grains, surface roughening on the free surface of the specimen was easy [37]. The crystal orientation between the grains caused the majority of the surface roughness in the metal microforming processes. Workpiece stress, caused by an applied load, resulted in both elastic and plastic strain during a forming process [41]. This is known as springback in sheet metal forming because of the elastic strain’s attempt to reclaim the deformed portion once external pressure has been removed [42,43]. The springback behaviour of a workpiece is directly influenced by its elasto-plastic properties. Springback is mostly caused by variations in grain orientation on a material’s surface [42]. A number of formation experiments have looked into springback behaviour. Another consideration in L-bending processes is that the springback effect has been observed to diminish with die-radius, die-angle, and punch-to-die clearance [44]. Furthermore, the springback angle in the rolling direction can be greater than the transverse angle in a rolling operation. The size influence on springback was studied by Gau et al. [43], using pure copper foils in three-point bending experiments. MST was used for all bending experiments [45,46].

This work described a method to design and develop a portable miniaturised UTM. The newly designed apparatus could achieve lateral displacements as comparatively small as 0.001 cm, and maximum loads of 0.1 kN. The mechanical properties of a 50 μm-thick copper foil were measured on this device and all results were compared to the average median results obtained from a commercialized Instron.

The reported results, and the performance of the developed test equipment, suggested that it was indeed a suitable device for the satisfactorily accurate measurement of the mechanical characteristics of thinner and softer materials.

Two pivotal benefits of this test measurement equipment were that it was cheaper to obtain and and slightly smaller in size than available commercialized equipment. Additionally, a significant characteristic of this equipment was the ease and convenience with which elements (load cell and fasteners) could be exchanged to meet user requirements. This new testing system can be used with a 3D laser-confocal microscope, allowing easy evaluation of the deformation behaviour of materials on a microscale in real-time.

## 5. Conclusions

Evaluation of local deformation behaviour in a very narrow region on a microscale could be a novel approach to exploring the impacts of size in metal microforming. In this work, a novel, small-scale engineering testing instrument for microscale specimens was developed that allowed in situ observations of the surface roughness and fracture mechanisms of materials by 3D laser-confocal microscope. This study focused on the development of surface roughness in the context of the cumulative influence of work-piece thickness and granular particulate size. The accompanying inferences depend upon reported outcomes:

**i.**  The surface roughness assessment on tensile-tested copper specimens showed the significant influences of size in metal microforming processes. It was concluded that the fracture-strain of pure copper foils was significantly diminished when the thickness changed from 0.3 to 0.05 mm, while the relative surface roughness of pure copper increased with decreases in foil thickness.
**ii.**  The fracture behaviour was mostly brought about by the concave surface formed by free surface roughening.
**iii.**  Findings with high stability can be discovered while moving with the sample grasped along the drive screw in graphical plots of the crosshead’s displacement against time. The results also revealed very low mechanical noise during displacement of the crosshead, meaning that there were no additional effects on the machine, such as vibrations or shifts in speed, that could influence performance.
**iv.**  The engineering stress-strain plots of the pure copper-tests with various thicknesses depicted a level of stress necessary to initiate plastic flowing inside the material. From these results, it was observed that strength and ductility declined with decreasing thickness. From the influence of thickness on fracture-strain, acquired during tensile-testing, it was clearly evident that the elongation-at-break of the pure copper foils decreased significantly with decreases in thickness.
**v.**  The relative average surface-roughness, R_a_, was evaluated, and it was evident that the surface-roughness escalated with the increasing trend of plasticity deformation (plastic-strain), ε. To allow better understanding of the effects of plastic strain on surface roughness prior to material fracture, micro tensile tests were performed on the newly-developed machine under a 3D laser-confocal microscope. It was clearly observed that homogeneous surface roughness was caused by plastic strain, which further formed the concave surface that led to fracture points. Finally, we also concluded that surface roughness was a crucial factor influencing the fracture behaviour of metallic sheet strips in metal microforming.
**vi.**  The developed testing instrument is also capable of testing ceramics, composites, and polymer-based microsamples. Moreover, with respect to biomedical engineering, newly-designed and developed bone plates’ fractures could be examined in detail with the help of this apparatus.

## 6. Future Work:

To understand the relationship between surface roughness and technical parameters such as grain size, a constitutive model should be generated to simulate surface evolution. Moreover, the new testing apparatus could be used with a 3D laser-scanning microscope, to evaluate the deformation behaviours of other materials and miniaturized components.

## Figures and Tables

**Table 1:** Typical micromanufacturing processes [5,6].

**Additive-processes**	Surface coating, microcasting, and sintering.
**Deforming-processes**	Microstamping, microforging, micro-deep drawing, and micro stamping.
**Hybrid-processes**	Laser-based microforming, and hybrid micromachining.
**Subtractive processes**	Micro-EDM, micro-ECM, and micro-EBM.

**Table 2:** Specifications of testing apparatus.

**Maximum stroke**	30 mm
**Configuration**	Twin column support
**Mounting**	Table/ground: Horizontal
**Load cell**	3 kg, 5 kg and 10 kg (as required)
**Weight**	5.5 kg (12 lbs)

**Table 3:** Chemical composition of pure copper (Cu), wt%.

**Element**	**Zn**	**Sn**	**Fe**	**P**	**Ni**	**Co**	**Pb**	**Cu**
**Contents**	0.01	<0.01	0.02	<0.003	0.02	0.03	0.02	>99.5

**Table 4:** Grain sample preparation process for microstructural analysis.

**Procedure**	**Surface**	**Time**	**Solution**
**Grinding**	9 μm Largo cloth	2 min	Water
**Polishing**	3 μm Mol cloth	1 min	Alpha aluminium powder (0.5 microns)
**Polishing**	OP-chem	20 s	Alpha aluminium powder (0.3 microns)

**Table 5:** Grain size of the specimens.

	**Sample 1**	**Sample 2**	**Sample 3**
**Material**	Copper	Copper	Copper
**Temperature**	700 °C	700 °C	700 °C
**Time**	10 min	10 min	10 min
**Thickness**	0.05 mm	0.1 mm	0.3 mm
**Average grain size**	0.052 mm	0.040 mm	0.075 mm
**Number of grains (nG)**	4.0	5.4	6.5
**Microstructure**	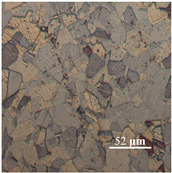	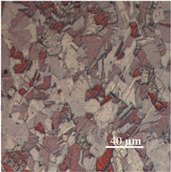	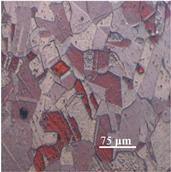

## Data Availability

The data presented in this study are available on request from the corresponding author.

## Abbreviations

LVDT	Linear-variable differential-transformers
AGI	Average-grain intercept
SSD	Statistically-stored dislocations
GND	Geometrically-necessary dislocations
UTM	Universal-testing machine
3D	Three-dimensional
T	Thickness
D	Grain-size
E	Young’s modulus
ε	Plastic-strain
𝜌	Density
Ҏ	Conventional effective plastic-strain
Ra	Arithmetic-average of the roughness-profile
V	Voltage
